# Products of Sericulture and Their Hypoglycemic Action Evaluated by Using the Silkworm, *Bombyx mori* (Lepidoptera: Bombycidae), as a Model

**DOI:** 10.3390/insects12121059

**Published:** 2021-11-25

**Authors:** Salvador D. Aznar-Cervantes, Beatriz Monteagudo Santesteban, José L. Cenis

**Affiliations:** Departamento de Biotecnología, Genómica y Mejora Vegetal, Instituto Murciano de Investigación y Desarrollo Agrario y Medioambiental (IMIDA), La Alberca, 30150 Murcia, Spain; bea.monteagudo1@gmail.com (B.M.S.); josel.cenis@carm.es (J.L.C.)

**Keywords:** fibroin, sericin, silkworm, chrysalis, diabetes

## Abstract

**Simple Summary:**

The use of invertebrates as animal models is gaining attention within the scientific community due to numerous advantages during the development of the experiments, low cost of rearing, and fewer ethical problems. The well-documented biology of the silkworm (*Bombyx mori*) makes this insect an ideal candidate to be used in different fields of research. In this study, we demonstrated the feasibility of using the silkworm to evaluate the hypoglycemic action of various products of sericulture included in the diet after promoting glucose or sucrose-induced hyperglycemia in silkworms. The postprandial antihyperglycemic activity of fibroin, sericin, and powder made from pupae of silkworms is confirmed. These natural products are therefore ideal candidates for the prevention and treatment of diabetes, obesity, and other lifestyle-related diseases.

**Abstract:**

Sericulture generates different natural products with potential medical applications. Silk peptides, worms, or even pupae are commonly employed in traditional Asian medicine with a wide variety of purposes, and some scientific work has been focused on their antidiabetic properties. This work evaluates the postprandial antihyperglycemic activity of fibroin, sericin, and powder made from either larvae or pupae of silkworms, and *Bombyx mori* L. (Lepidoptera: Bombycidae), employing the silkworm itself as an animal model. The results indicate a reduction in the glucose levels in hemolymph after sucrose or glucose-induced hyperglycemia when these products are included in the diet of the worms.

## 1. Introduction

The growing number of studies targeting natural compounds to treat various pathologies suggests the importance of this field of research within the scientific community. Traditional Asian medicine has inspired many of these studies, being a rich source of ideas in exploring new bioactive compounds. Sericulture is a production cycle that involves the use of mulberry to feed the worms until the cocoon is spun by the larvae. Subsequently, the silk is degummed, and it is used for the textile industry or some incipient biomedical applications. Throughout this process, several potential products of medical interest are generated. This is not surprising, given the multiple applications attributed to the silkworms or the silk proteins (fibroin and sericin) in Asia. On the one hand, the powder produced from dehydrated silkworm chrysalides (pupae) has demonstrated potential medical uses presenting notable activities, such as increasing fat metabolism in rats [1], increasing levels of nitrite and nitric oxide synthase expression in a model of erectile dysfunction in rats [2], inducing apoptosis in human gastric cancer cells [3], and constituting a healthy nutritional source of protein and fat [4,5]. On the other hand, the powder made from silkworm larvae induces a reduction of plasma glucose level [6,7] due to the presence of 1-deoxynojirimycin (DNJ) ingested and accumulated during the feeding with mulberry leaves. DNJ presents α-glucosidase inhibitory effect [8], and the silkworm powder also inhibits the expression of glucose transporter (SGLT1) of human intestinal epithelial cell line Caco-2 [9]. Moreover, silk proteins present interesting biomedical properties when administered as nutritional supplements. An increase in fat oxidation and exercise performance has been demonstrated in mice after ingesting silk peptides in the diet [10,11]. Furthermore, fibroin, sericin or peptides, and hydrolysates derived from cocoons also present α-glucosidase inhibitory activity as previously stated in several studies [6,12,13,14].

The use of invertebrates as animal models is gaining attention within the scientific community due to several advantages in the design and execution of the experiments, low cost of rearing, and fewer ethical problems. The well-documented biology of the silkworm (*B. mori*), as well as its ancestral domestication, make this insect an ideal candidate to be used as an animal model in different fields of research. In fact, its utility as a model for evaluating antidiabetic drugs has already been investigated [15,16], as well as its use in the study of the pathogenicity of bacteria [17,18], fungi [19], and drugs for the treatment of different pathologies [20,21]. In this context, this work aims to demonstrate the feasibility of using the silkworm to evaluate the hypoglycemic action of various products of sericulture by including them in the diet after promoting glucose or sucrose-induced hyperglycemia in silkworms. As far as we know, this is the first time that the silkworm is used as a model to evaluate the products derived from its rearing for the potential use in the prevention or treatment of diabetes.

## 2. Materials and Methods

### 2.1. Silkworm Rearing

A Spanish hybrid of silkworm races (*Sierra Morena X Bagdad)* was used for this experiment. The larvae were fed until the first day of the fifth instar with an artificial diet provided by the Padua Sericulture Station [22] at 23–25 °C and 50–60% relative humidity, under the normal photoperiod in our location in Spain during August (14 h light:10 h darkness). These worms were subsequently used to induce the sucrose or glucose-derived hyperglycemia (except the negative control) and to administer the different products in the same hyperglycemic diets.

### 2.2. Fibroin and Sericin Extraction

The purification of silk fibroin involved degumming in order to remove the sericin. White cocoons were boiled for 30 min in an aqueous solution of 0.02 M Na_2_CO_3_ (Panreac, Barcelona, Spain) and then rinsed thoroughly with distilled water to extract the glue-like sericin proteins. The extracted fibroin was then dried at room temperature for three days and dissolved in LiBr (Acros Organics, Fairlawn, NJ, USA) 9.3 M for 3 h at 60 °C to generate a 20% *w*/*v* solution. Then it was dialyzed against distilled water for three days (performing at least eight water changes). The resultant aqueous solution was lyophilized in order to have purified fibroin ready to be dissolved in the water employed to prepare the diets prior to feeding the worms [23].

The extraction of silk sericin was performed by autoclaving the cocoons in distilled water (25 g/L) at 120 °C for 1 h [24] to obtain pure sericin aqueous solutions after filtering them. Then, these solutions were freeze-dried, obtaining a sericin powder ready to be included in the diets. Both sericin and fibroin were stored dry at room temperature until use.

### 2.3. Preparation of Silkworm and Chrysalis Powder

Prior to hyperglycemic induction experiments, the first batch of worms (N = 50) was fed ad libitum with mulberry leaves to enrich silkworm larvae and pupae with the components derived from the foliage and DNJ, among others. Part of the fifth instar larvae (N = 25) was frozen and subsequently freeze-dried; the same procedure was performed using chrysalides (N = 25). The entire body of lyophilized larvae or pupae was ground using a waring^®^ commercial laboratory blender and incorporated as a supplement to the artificial diet, as detailed below. Both powders were stored in a dry container at room temperature, protected from light until use.

### 2.4. Induction of Hyperglycemia and Treatment with Different Products

**Figure 1:**
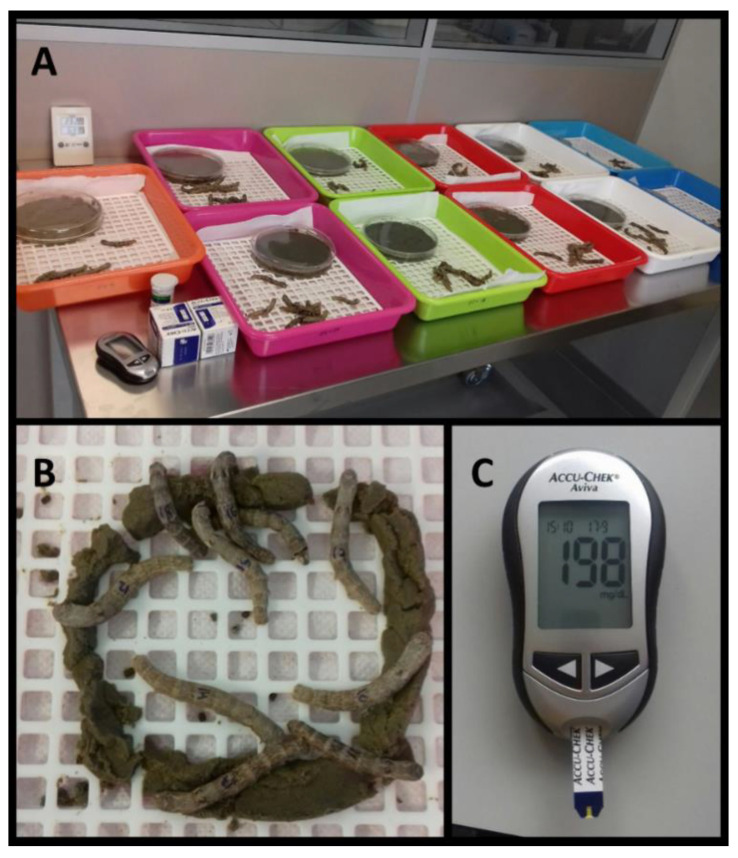
Illustrative images of the different batches of worms with their respective diets prepared immediately before feeding (**A**), silkworms actively feeding (**B**) and example of measurement of glucose levels in the hemolymph (**C**).

Eleven types of diet were prepared to feed worms from the eleven different batches (N = 10) during 24 h ad libitum (Figure 1). After this period, the glucose levels in hemolymph were determined, as explained below.

The negative control diet (considered normal) was prepared by mixing 25 g of powdered diet, supplied by the Padua Sericulture Station, with 75 mL of boiling water (ratio 1:3) until a consistent homogeneous paste was obtained that was allowed to cool down before use.

Following a similar procedure, two types of hyperglycemic diets (considered positive controls) were prepared separately, one including glucose and the other sucrose (Sigma-Aldrich, St. Louis, MO, USA), both at 10% (*w*/*w*) as previously stated by several authors [25]. Briefly, glucose or sucrose was dissolved in the water that would later be used to add and mix the powdered diet.

In order to evaluate the potential postprandial antihyperglycemic activity of fibroin, sericin, and powder made from either larvae or pupae of silkworms, these four products were added at 5% (*w*/*w*) to eight different diets, four containing glucose and four containing sucrose, at 10% (*w*/*w*) in both cases, comparable to the diets considered positive controls of hyperglycemic induction. Sericin and fibroin were dissolved in the water of their respective diets prior to mixing them with the commercial powdered diet. On the other hand, the silkworm or chrysalis powder was directly homogenized in the diet.

### 2.5. Evaluation of Glucose Level in Haemolymph

Glucose levels in the hemolymph were determined immediately 24 h after the start of the feeding as described by other authors [16,25], collecting it from the silkworms through a cut on the first proleg, making this determination in at least three specimens per treatment and using a glucometer (Accu-Check, Roche, Basel, Switzerland) for this purpose. Briefly, the glucometer test strips are filled by capillary action when they come into contact with the drop of hemolymph produced after making the incision, without the need to carry out any type of volumetric measurement or dilution of the sample. The choice of the feeding period is based on previous experiences in our laboratory, in which we confirmed that it is an adequate time to establish high glucose levels in the hemolymph.

### 2.6. Statistical Analysis

For the statistical analyses, SPSS 25 software was used. When the data were compiled with the normality and homogeneity of variance requirements, they were compared using parametric tests: ANOVA followed by Bonferroni’s post hoc multiple *t*-test. When the assumption of homoscedasticity was not satisfied, the statistical significance was determined using Mann–Whitney test (nonparametric). In every situation, the significance level was set to *p* < 0.05.

## 3. Results and Discussion

### 3.1. Effective Induction of Hyperglycemia in Silkworms Using Glucose or Sucrose as Additives in the Artificial Diet

**Figure 2:**
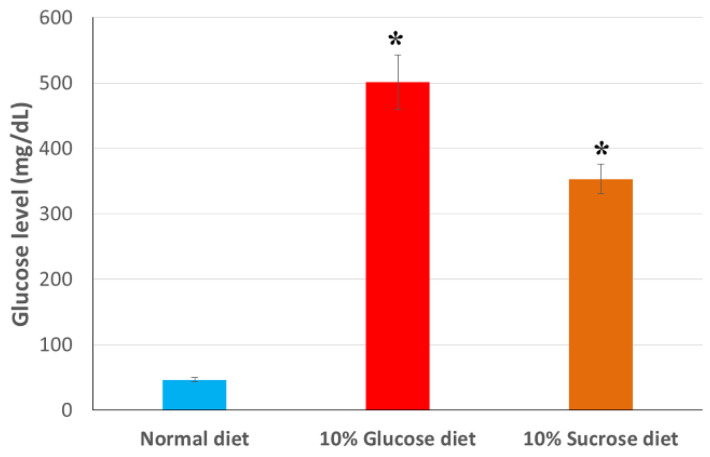
Glucose levels in hemolymph detected in silkworms fed a standard artificial diet (regular diet) and diets enriched in glucose or sucrose (10% *w*/*w*) 24 h after the start of the feeding. Data are expressed as mean ± SEM. * Indicates significantly higher values compared to the control diet (*p* < 0.05).

As expected, feeding silkworms with an artificial diet enriched with glucose or sucrose at 10% (*w*/*w*) significantly increased glucose levels in the hemolymph (Figure 2). These differences were statistically significant in both comparisons (Bonferroni, *p* < 0.05), with the negative control (standard diet), and between both hyperglycemic treatments. The mean value obtained in the case of worms fed using a diet enriched in glucose (501 mg/dL) was higher than that using diet enriched in sucrose (353 mg/dL). Moreover, both are far superior to the average value of the negative control diet (46 mg/dL). These hemolymph glucose levels are consistent with those reported in similar studies after inducing hyperglycemia by feeding sucrose or glucose-enriched diets, consistently higher in the latter case [15,25,26].

### 3.2. Hypoglycemic Action of Products Derived from Sericulture under Glucose-Induced Hyperglycemia

As it has been previously stated, there is a similar mechanism, in terms of uptake and storage of sugars, in silkworms and mammals, displaying common features that make feasible the use of silkworm models for the assessment of antidiabetic drugs for both types of diabetes (I and II) [26]. In this work, two types of hyperglycemia have been induced in silkworms; on the one hand, using a diet enriched in glucose, and on the other, employing sucrose. This helps us discern, at least partly, whether the mechanism of action of this potential hypoglycemic effect is related to inhibition of glucose absorption, or on the contrary, is more linked to inhibition of α-glucosidase activity.

**Figure 3:**
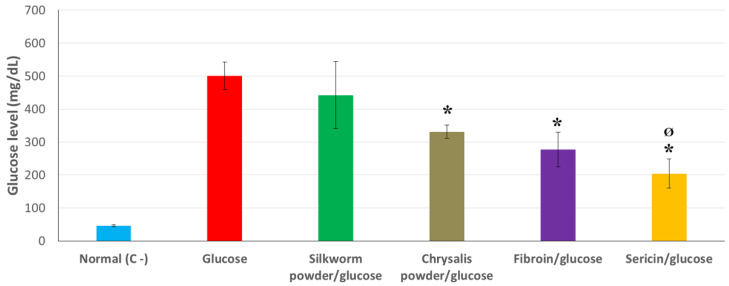
Glucose levels in hemolymph detected in silkworms fed a standard artificial diet (regular diet), a diet enriched in glucose (10% *w*/*w*), and different diets containing the same amount of glucose and the products studied at 5% *w*/*w* (silkworm powder, chrysalis powder, fibroin or sericin). Determinations were carried out 24 h after the start of feeding. Data are expressed as mean ± SEM. * Indicates values significantly lower than the glucose diet considered positive control for induction of hyperglycemia (*p* < 0.05). **^Ø^** Indicates values equivalent to the negative control of hyperglycemia (regular diet) considered as a nonpathological condition (*p* > 0.05).

In this sense, the data obtained from the experiment using glucose (monosaccharide) revealed a hypoglycemic effect in all the treatments studied, decreasing the average values of glucose. These values were significantly lower than those obtained in worms fed the positive control diet in the case of feeding with chrysalis powder, fibroin, or sericin (*p* < 0.05). In this last case recovering glucose values that were statistically equivalent to the negative control, which simulates a nonpathological condition (Bonferroni, *p* > 0.05). Figure 3 clearly shows a larger SEM in the glucose values of silkworms fed with worm powder (442 ± 102 mg/dL). This result could be due to the lower homogeneity in terms of its distribution in the artificial diet. It was observed that the powder resulting from the grinding of the worms, and its distribution in the diet, were more heterogeneous than in the case of the chrysalis. This fact could lead to a differential intake of this product by the different specimens, thus increasing the variability in the results. Similarly, previous work that compared the antihyperglycemic effect of worm powder, fibroin, and sericin using rats as an animal model, obtaining better results with fibroin or sericin than with worm powder [6]. This result is analogous to the findings in our study us using *B. mori* as an animal model. Furthermore, silkworm powder has been described as an inhibitor of the expression of glucose transporter (SGLT1) of human intestinal epithelial cell line Caco-2 [9], and this type of transporters are sometimes detected in insects [27]. Therefore, it can be suggested that part of the hypoglycemic effect, in this case, could be due to this mechanism, both for worm and chrysalis powder. However, this aspect should be further studied to confirm this analogy.

Silk peptides have been studied previously due to their antidiabetic properties. Some studies have shown, for example, how the consumption of silk hydrolysates in rats promotes improvements in antidiabetic symptoms by potentiating insulin secretion [28]. Taking into account the presence of a peptide hormone in the silkworm, similar in structure to the insulin, called bombyxin, as well as the existence of an insulin signaling pathway it activates with the same mode of action as that of mammals [16], we can begin to understand at least part of the mechanism involved in the decrease in the hemolymph glucose values detected in our experiment, especially when fibroin or sericin were used in the artificial diet. It is important to mention that hydrolyzed silk fibroin also promotes the regeneration of pancreatic β-Cells, as stated by Park et al. [29].

### 3.3. Hypoglycemic Action of Products Derived from Sericulture under Sucrose-Induced Hyperglycemia

Sucrose is a sweetener widely used in foods and beverages whose abuse can lead to postprandial hyperglycemia, which in the long term implies the development of diseases such as obesity or diabetes [30]. Therefore, it is important to minimize these effects of the blood glucose caused by sucrose and thus avoid the emergence of these lifestyle-related diseases [15]. Sucrose is divided into glucose and fructose under the action of the enzyme α-glycosidase in the intestine to be absorbed, subsequently causing undesirable increases in blood glucose [31]. Thus, the inhibition of the catalytic activity of this enzyme is important for the prevention and treatment of diabetes, and several compounds used as antidiabetic drugs are based on precisely this inhibitory mechanism (acarbose and voglibose, among others).

**Figure 4:**
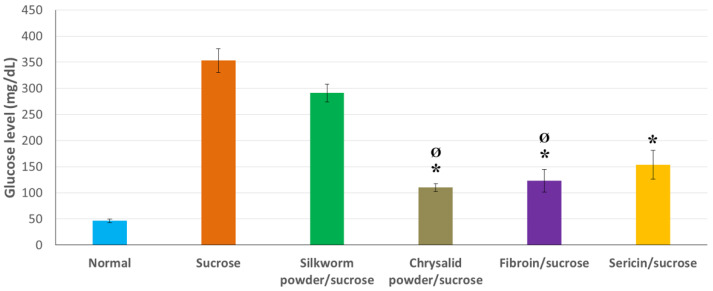
Glucose levels in hemolymph detected in silkworms fed a standard artificial diet (regular diet), a diet enriched in sucrose (10% *w*/*w*), and different diets containing the same amount of sucrose and the products studied at 5% *w*/*w* (silkworm powder, chrysalis powder, fibroin or sericin). Determinations were carried out 24 h after the start of feeding. Data are expressed as mean ± SEM. * Indicates values significantly lower than the sucrose diet considered positive control for induction of hyperglycemia (*p* < 0.05). **^Ø^** Indicates values equivalent to the negative control of hyperglycemia (regular diet) considered as a nonpathological condition (*p* > 0.05).

Figure 4 shows the average values of glucose obtained from the hemolymph of worms fed different diets. In this case, the induction of hyperglycemia was carried out by adding sucrose at 10% (*w*/*w*) to both the positive control diet and the diets including the study products, the latter added at 5% *w*/*w* (explained in the Section 2). Furthermore, glucose measurements were also conducted in worms fed a standard artificial diet, named regular diet, and those considered negative control of hyperglycemia. Sucrose is a disaccharide divided in the intestines into glucose and fructose, its two basic units, to allow the absorption of this glucose later and finally raise its levels in hemolymph in a way analogous to what that occurs in human blood. The fact that this increase in hemolymph glucose levels varies after feeding with sucrose-enriched diets and different compounds would suggest varying degrees of inhibition of the α-glucosidase activity (respectively) since this enzyme is responsible for the breakdown of the disaccharide towards its component monosaccharides.

As shown in Figure 4, the trend in terms of the hypoglycemic effect of the different products studied was similar to that obtained in the case of glucose-induced hyperglycemia (Figure 3) with some variations. Once again, the silkworm powder showed a certain reduction in the average glucose value, but this was not detected as statistically significant with respect to the positive control of hyperglycemia in the sucrose diet (Bonferroni, *p* > 0.05). On the other hand, significantly lower glucose values were detected in the case of feeding with chrysalis powder, fibroin, and sericin compared with the positive control (Bonferroni, *p* < 0.05). These values were equivalent to the negative control diet (normal diet) in the case of chrysalis powder and fibroin (Bonferroni, *p* > 0.05). Therefore, both are the most effective candidate products in the approach of hyperglycemia induced feeding with sucrose.

The inhibitory effect of α-glucosidase activity in silkworm powder obtained from larvae has been previously stated [7,8,9], and it is related to its high content in DNJ, even higher than that of mulberry leaves on which they feed [8]. Therefore, it would accumulate to a greater extent the longer the worm spends feeding throughout its life cycle. Hence, the hypoglycemic effect is more significant in the case of chrysalis powder. Moreover, this inhibition of α-glucosidase activity has also been indicated for sericin [14,32] and fibroin [13], with fibroin reporting a potential regenerative effect of pancreatic cells [29].

## 4. Conclusions

In this work, we demonstrated the feasibility of using the silkworm to evaluate the hypoglycemic action of various products resulting from sericulture by including them in the diet after promoting glucose or sucrose-induced hyperglycemia. As far as we know, this is the first time that the silkworm is used as a model to evaluate the products derived from its rearing in relation to the potential use in the prevention or treatment of diabetes.

The postprandial antihyperglycemic activity of fibroin, sericin, and powder made from pupae of silkworms (*B. mori*) is confirmed after its addition to hyperglycemic artificial diets. The inhibitory effect of α-glucosidase activity, confirmed in these products [7,8,9,12,13,14,32], as well as other previously described mechanisms, such as the inhibition of the expression of intestinal glucose transporters [9], or even the activation of insulin-like signaling pathway by different peptides derived from silk [28], seem to be responsible for this interesting hypoglycemic effect. Therefore, these products derived from sericulture could be ideal candidates for the prevention and treatment of diabetes, obesity, and other lifestyle-related diseases.

## Data Availability

Not applicable.
